# Tissue IL-6/LIF/LIFR and CXCL9 Expression Correlates with High-Risk NBI Patterns and Squamous Cell Carcinoma in Vocal Fold Lesions

**DOI:** 10.3390/ijms27041923

**Published:** 2026-02-17

**Authors:** Magda Barańska, Katarzyna Taran, Wioletta Pietruszewska

**Affiliations:** 1 Department of Otolaryngology, Head Neck Oncology, Medical University of Lodz, 90-153 Lodz, Poland; 2 Department of Pathomorphology, Chair of Oncology, Medical University of Lodz, 92-213 Lodz, Poland

**Keywords:** laryngeal cancer, narrow band imaging, interleukin 6, leukemia inhibitory factor, leukemia inhibitory factor receptor, C-X-C motif chemokine ligand 9, biomarkers

## Abstract

Laryngeal squamous cell carcinoma (SCC) remains a major clinical challenge due to substantial mortality and limited preoperative risk stratification. Narrow-Band Imaging (NBI) enables real-time visualization of mucosal microvasculature, yet the molecular correlates of high-risk NBI phenotypes in vocal fold lesions are incompletely defined. In a prospective cohort of 145 patients with vocal fold lesions, NBI microvascular patterns were graded using the Ni classification and dichotomized using a pre-specified high-risk threshold (Ni ≥ 4 vs. Ni ≤ 3). Histopathology was classified according to WHO 2017. Epithelial expression of IL-6, LIF, LIFR and CXCL9 was quantified by immunohistochemistry using the immunoreactive score (IRS). Associations were tested using non-parametric methods and logistic regression, and diagnostic performance was assessed by ROC analysis. SCC was diagnosed in 63/145 cases. The Ni category showed a strong stepwise association with WHO 2017 histopathological severity. Using Ni ≥ 4, diagnostic performance for SCC was balanced (sensitivity 82.5%, specificity 82.9%; accuracy 82.8%). LIF and LIFR expression decreased with increasing histopathological severity and higher-NBI-risk categories, whereas CXCL9 increased with more suspicious NBI patterns; epithelial IL-6 did not differ across lesion categories. In multivariable logistic regression, Ni ≥ 4 was the strongest independent predictor of SCC (adjusted OR 8.90), while higher LIF (adjusted OR 0.73) and LIFR (adjusted OR 0.78) were independently associated with lower odds of SCC (model AUC 0.943). Multivariable analysis confirmed NBI as the strongest independent predictor of carcinoma, while epithelial LIF and LIFR expression showed inverse associations with histological malignancy and high-risk NBI vascular patterns. LIF/LIFR and CXCL9 show distinct, biologically plausible associations with NBI risk phenotypes, suggesting that selected tissue markers may complement NBI for refined SCC risk stratification.

## 1. Introduction

Laryngeal squamous cell carcinoma (LSCC) is the main histological subtype of malignant laryngeal tumors and remains a challenge in head and neck oncology [1]. Globally, LSCC accounted for approximately 189,191 new cases and 103,359 deaths in 2020 and reflects a mortality-to-incidence ratio exceeding 50% [2]. While early glottic lesions are often detected due to symptomatic hoarseness, presenting a favorable 5-year survival rate of 82.6% [3] supraglottic and subglottic tumors typically present at more advanced stages, resulting in considerably poorer prognoses. Despite advances in surgical interventions, endoscopic visualization, and radiotherapy techniques, the overall 5-year survival rate for LSCC remains near 60%, largely due to high recurrence rates and late-stage diagnosis [4]. Laryngeal squamous cell carcinoma is characterized by early and progressive remodeling of the superficial mucosal microvasculature, driven by epithelial–stromal interactions, inflammatory signaling, immune modulation, and extracellular matrix reorganization. This highlights a necessity for biomarkers that not only improve early detection but also predict tumor behavior.

Angiogenesis is a hallmark of cancer, providing tumors with essential oxygen and nutrients for lesions larger than 1–2 mm in diameter, and facilitating invasion and metastasis [5]. In LSCC, the molecular determinants of microvascular remodeling remain incompletely defined [6]. Narrow-Band Imaging (NBI) enhances visualization of superficial mucosal vessels and enables real-time evaluation of microvascular architecture. In the Ni et al. (2011) classification, microvascular patterns are graded from type I to V, with progressively more irregular and perpendicular intraepithelial papillary capillary loops (IPCLs) indicating increasing risk of dysplasia and squamous cell carcinoma; this risk gradient has been supported across multiple clinical studies in laryngeal lesions [7,8,9]. The vascular patterns visualized by NBI, therefore, represent the morphological manifestation of underlying biological processes rather than direct visualization of neoplastic cells, and while NBI improves diagnostic precision, the molecular correlates of these endoscopically visible vascular phenotypes remain insufficiently characterized. Consequently, NBI findings reflect the integrated biological output of multiple molecular pathways rather than isolated epithelial alterations. In this context, tissue-based immunohistochemistry does not serve as a diagnostic alternative to NBI but enables exploration of molecular mechanisms that may underlie specific vascular phenotypes.

Emerging evidence indicates that interleukin-6 (IL-6), leukemia inhibitory factor (LIF), its receptor (LIFR), and the chemokine CXCL9 are involved in regulating inflammation, angiogenesis, and immune modulation within the tumor microenvironment of head and neck squamous cell carcinomas [10,11,12,13]. IL-6 is a multifunctional cytokine that activates the JAK/STAT3 pathway, promoting cell proliferation, resistance to apoptosis, angiogenesis, and metastasis [14]. In LSCC, elevated IL-6 levels are associated with aggressive tumor behavior, treatment resistance, and recurrence, in part through upregulation of VEGF and modulation of tumor–stromal interactions [15,16]. Importantly, tissue immunohistochemistry primarily reflects compartment-specific protein abundance; therefore, epithelial IL-6 immunoreactivity may not capture the predominantly stromal/endothelial IL-6 trans-signaling that drives STAT3 activation and angiogenic remodeling within the tumor microenvironment [14]. LIF, a member of the IL-6 cytokine family, signals through LIFR and gp130 to activate STAT3, MAPK, and PI3K/AKT pathways, promoting epithelial–mesenchymal transition, maintenance of cancer stem-like cells, and immune evasion [17,18,19].

Several studies investigating LIF and LIFR expression in oncology have produced contradictory results, reporting both pro-tumorigenic and anti-tumorigenic effects. This complexity reflects the various roles of LIF/LIF-R signaling, which can activate MAPK, JAK/STAT3, and PI3K/AKT pathways, influencing differentiation, stem cell maintenance, survival, and immune responses. A recent study indicates that LIF levels are reduced in oral squamous cell carcinoma compared with normal, while LIFR is upregulated, suggesting that LIF may be linked to cellular differentiation, whereas LIFR may contribute to tumor progression [20]. This observation is consistent with the concept of “unlocking phenotypic plasticity” in cancer, where loss of differentiation is associated with more aggressive behavior [17,20,21]. Beyond differentiation-related effects, LIF/LIFR signaling has been linked to ERK/Akt-associated EMT programs and extracellular matrix remodeling, processes that are tightly coupled to angiogenesis and may therefore relate to the vascular phenotypes observed on NBI [22].

CXCL9, an interferon-γ-inducible chemokine, is responsible for the recruitment of CXCR3+ effector T cells and natural killer cells, regulating the immune landscape of tumors. Its expression can both reflect effective anti-tumor immunity and, under chronic inflammatory or immune-edited conditions, contribute to immune tolerance and T-cell exhaustion [23]. These molecules collectively define two complementary axes in LSCC: the IL-6/LIF/LIFR-driven pro-oncogenic and angiogenic axis and the CXCL9-mediated immune surveillance axis, which together may determine malignant potential and recurrence risk [13,18,24]. Immune mediators, particularly interferon-inducible chemokines such as CXCL9, contribute not only to immune-cell recruitment but also to endothelial activation and vascular remodeling. Such immune–vascular interactions may directly influence the morphology of superficial mucosal capillaries visualized by NBI.

Despite increasing interest in these pathways, prior studies have primarily focused on serum cytokine levels, overlooking tissue-level expression and its direct correlation with histopathology or endoscopic vascular features. To our knowledge, no previous study has simultaneously assessed the expression of IL-6, LIF, LIFR, and CXCL9 in laryngeal lesions and correlated these findings with NBI vascular patterns. Understanding these relationships could provide insights into the molecular mechanisms underlying neovascularization, immune modulation, and malignant transformation in LSCC. Moreover, integrating molecular profiling with endoscopic imaging has the potential to enhance early diagnostic accuracy, inform risk stratification, and guide individualized therapeutic approaches.

This study aimed to characterize epithelial tissue expression (IRS) of IL-6, LIF, LIFR and CXCL9 across vocal fold lesions categorized by WHO 2017 histopathology (non-dysplastic epithelium, LGD, HGD and squamous cell carcinoma) and to investigate whether epithelial expression of IL-6, LIF, LIFR, and CXCL9 correlates with NBI-defined microvascular patterns and histopathological severity in vocal fold lesions. Rather than proposing biomarkers as alternative diagnostic tools, the study sought to explore molecular pathways potentially underlying NBI-visible vascular phenotypes. The biomarkers selected for this study were not intended to represent the full spectrum of inflammatory or angiogenic mediators but were chosen to capture key biological axes implicated in epithelial–stromal interactions and microvascular remodeling in laryngeal carcinogenesis.

Tissue-level expression of cytokines represents a functional readout of epithelial signaling pathways that regulate endothelial behavior indirectly through paracrine mechanisms, stromal activation, and extracellular matrix remodeling. Alterations in these pathways can translate into changes in capillary density, orientation, and loop morphology, which are visualized endoscopically as characteristic NBI vascular patterns.

We hypothesized that IL-6 expression would be associated with high-risk NBI patterns and with squamous cell carcinoma. We further assessed whether combining NBI findings (including the pre-specified Ni ≥ 4 high-risk threshold) with the IL-6/LIF/LIFR axis and CXCL9 improves tissue-level risk stratification for squamous cell carcinoma. As an exploratory objective, we examined whether IRS marker expression varies with SCC differentiation grade (G1–G3) as a surrogate of tumor aggressiveness.

## 2. Results

### 2.1. Patient Cohort and Clinicopathological Characteristics

A total of 145 patients (93 males, 52 females; mean age: 65.3 ± 11.7 years, range 35–89) with vocal fold lesions were included. The most frequent clinical presentations were tumors (*n* = 71), leukoplakia (*n* = 29), Reinke’s edema (*n* = 22), and polyps (*n* = 23). Histopathological diagnoses comprised the following categories: benign lesions—non-dysplastic epithelium (*n* = 59); premalignant lesions—low-grade dysplasia (*n* = 11) and high-grade dysplasia (*n* = 12); and malignant lesions—squamous cell carcinoma (*n* = 63) [25]. Most procedures were performed using transoral laser microsurgery (TOLMS) (*n* = 127), with an additional 18 total laryngectomies. The characteristics of the study group are presented in Table 1.

Clinical lesion type at presentation (e.g., benign: vocal fold polyp, Reinke’s edema, premalignant: leukoplakia, tumor/exophytic mass) was recorded based on white-light endoscopic appearance to characterize the cohort and reflect real-world referral phenotypes. Importantly, these categories do not constitute histopathological diagnoses. Therefore, all inferential analyses and outcome definitions were based on the WHO 2017 histopathological classification, while clinical lesion type was reported descriptively (Table 2).

### 2.2. Endoscopic Findings (WLI and NBI Evaluation)

#### 2.2.1. NBI Classification Patterns

In total, 145 laryngeal lesions were classified according to the WHO 2017 system as normal epithelium in 59/145 (40.7%), low-grade dysplasia (LGD) in 11/145 (7.6%), high-grade dysplasia (HGD) in 12/145 (8.3%), and squamous cell carcinoma (SCC) in 63/145 (43.4%).

There was a strong association between NBI Ni category (Ni2-Ni5) and histopathology (χ^2^(9) = 138.05, *p* = 2.6 × 10^−25^; Cramér’s V = 0.56), with a clear stepwise increase in SCC prevalence across Ni classes: 6/67 (9.0%) for Ni2, 5/12 (41.7%) for Ni3, 11/20 (55.0%) for Ni4, and 41/46 (89.1%) for Ni5 (Cochran–Armitage trend Z = 8.49, *p* = 2.12 × 10^−17^).

Using Ni2 as the reference, the odds of SCC were higher in Ni3 (OR = 7.26; 95% CI 1.75–30.09), Ni4 (OR = 12.43; 95% CI 3.68–41.93) and particularly Ni5 (OR = 83.37; 95% CI 23.86–291.30). When applying a clinically pragmatic cut-off of Ni ≥ 4 (Ni4 + Ni5) for SCC detection, the 2 × 2 table yielded TP = 52, FP = 14, FN = 11 and TN = 68, corresponding to a sensitivity of 82.5% (52/63), specificity of 82.9% (68/82), PPV of 78.8% (52/66), NPV of 86.1% (68/79), and overall accuracy of 82.8% (120/145). Notably, the Ni3 subgroup demonstrated a high proportion of advanced pathology, with HGD present in 6/12 (50.0%) and SCC in 5/12 (41.7%), i.e., HGD + SCC in 11/12 (91.7%), consistent with the concept of an “umbrella effect” in leukoplakia potentially obscuring vascular patterns and masking high-grade disease within lesions classified as type 3 (Table 3).

#### 2.2.2. Reproducibility of NBI Assessment

Inter-observer agreement for NBI vascular pattern assessment was consistently high. The unweighted Cohen’s κ reached 0.929 (95% CI: 0.877–0.981), indicating strong concordance. When accounting for the ordinal structure of the 1–5 Ni scale, the quadratic-weighted κ increased to 0.969 (approximate 95% CI: 0.94–0.99). The overall proportion of identical ratings between observers was 95.2%. Intra-observer reproducibility was similarly high. The unweighted Cohen’s κ was 0.950, reflecting a high degree of consistency between the two rating sessions. Weighted analyses further strengthened these results: the linear-weighted κ was 0.976, and the quadratic-weighted κ reached 0.990. The overall agreement between the two assessments was 96.6%.

Similar levels of interobserver agreement (κ approximately 0.75–0.90) have been reported in previous NBI studies of laryngeal lesions performed by experienced observers, indicating that the reproducibility observed in the present cohort is consistent with existing literature [26].

#### 2.2.3. Diagnostic Accuracy of the Ni ≥ 4 Threshold for SCC

Using Ni ≥ 4 (Ni4–Ni5) as a positive endoscopic threshold for SCC, the combined table summarizes both the underlying 2 × 2 classification and the resulting diagnostic performance (N = 145). The contingency components showed 52 true positives, 14 false positives, 11 false negatives, and 68 true negatives, corresponding to 63 SCC and 82 non-SCC cases overall. This threshold demonstrated a sensitivity of 82.5% (52/63) and a specificity of 82.9% (68/82), indicating a balanced ability to correctly identify SCC and non-SCC lesions. The positive predictive value (PPV) was 78.8% (52/66), whereas the negative predictive value (NPV) was 86.1% (68/79), suggesting that a negative result (Ni ≤ 3) reliably reduced the likelihood of SCC in this cohort. Overall accuracy was 82.8% (120/145). Likelihood ratios further supported the clinical utility of this cut-off: LR+ 4.83 indicates a moderate increase in the post-test probability of SCC when Ni ≥ 4 is observed, while LR− 0.21 reflects a substantial reduction in SCC probability when the test is negative (Table 4).

Odds ratios were calculated for SCC (yes/no) comparing each Ni category with Ni2 as the reference group. For clinical decision-making, Ni grades were also dichotomized using a single pre-specified cut-off: Ni ≥ 4 (Ni4–Ni5) versus Ni ≤ 3 (Ni2–Ni3) (Table 5). No Ni type 1 lesions were observed; therefore, Ni ≤ 3 corresponds to Ni 2–3 in this cohort.

### 2.3. Immunohistochemical Expression of IL-6, LIF, LIFR, and CXCL9

IRS values varied significantly among examined categories, in compliance with the WHO Classification of Tumours (Blue Book) for LIF, LIFR, and CXCL9, whereas IL-6 did not display significant variation (Table 6) [25]. For LIF, a clear decline in median expression was observed with increasing histological severity (Benign 8.0, LGD 6.0, HGD 4.0, SCC 2.0; Kruskal–Wallis *p* < 0.001). Post hoc pairwise testing (Mann–Whitney U with Bonferroni correction) demonstrated significant differences between benign tissue and HGD (*p* = 0.0322), between benign tissue and SCC (*p* < 0.001), and between LGD and SCC (*p* = 0.0156).

For LIFR, the median expression was high in benign and LGD lesions (8.0 and 9.0, respectively) but markedly lower in HGD and SCC (3.5 and 4.0; Kruskal–Wallis *p* < 0.001). Post hoc testing confirmed significant differences between benign tissue and HGD (*p* = 0.0006) and between benign tissue and SCC (*p* < 0.001), with a borderline difference between LGD and SCC (*p* = 0.0642). CXCL9 expression differed across categories (Kruskal–Wallis *p* = 0.0078) and was higher in SCC compared with benign lesions (post hoc *p* = 0.004). No significant differences in IL-6 expression were observed among the WHO categories (*p* = 0.4412). Shapiro–Wilk testing indicated non-normal distributions for all markers; therefore, non-parametric methods were applied.

### 2.4. Inter-Observer Reliability for IRS

IRS scoring was performed independently by two blinded observers. The intraclass correlation coefficient (ICC 2,1) for continuous IRS values across all four markers was 0.91 (95% CI 0.88–0.94), indicating excellent agreement between observers and confirming that the scoring method is both reliable and reproducible.

### 2.5. Representative Immunohistochemistry Images

**Figure 1:**
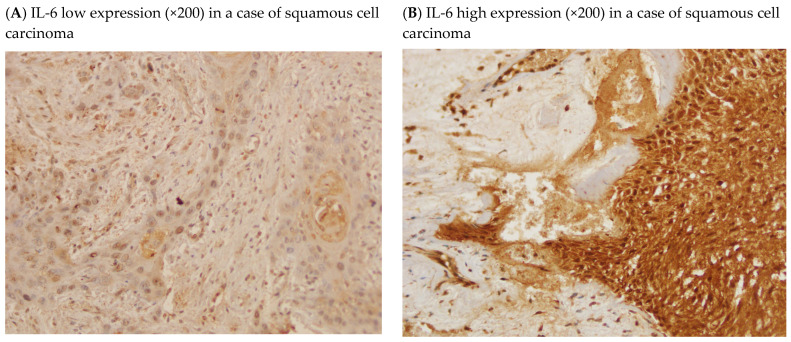

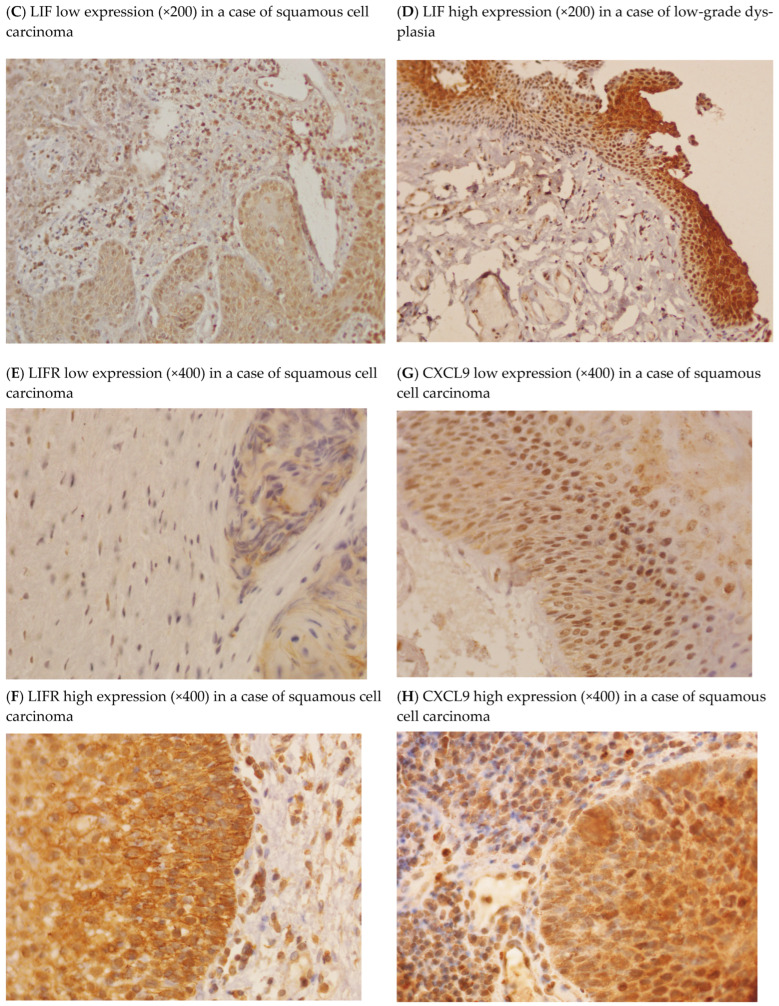
Representative IHC images illustrating the expression levels of IL-6, LIF, LIFR, and CXCL9.

Figure 1A–H provides representative examples of low and high immunoreactivity for each marker (IL-6, LIF, LIFR, and CXCL9) at ×200 and ×400 magnification. These images illustrate the range of staining intensities and distribution patterns that formed the basis of IRS evaluation.

### 2.6. Influence of Sex and Age

Age differed significantly across WHO 2017 histopathological categories (Kruskal–Wallis *p* = 4.7 × 10^−7^), showing a stepwise increase in median age from benign lesions (median 59 years), through LGD (68 years) and HGD (71 years), to SCC (72 years) (Table 7). In pairwise comparisons, patients with SCC were older than patients with non-SCC lesions overall (descriptive), supporting age as an important demographic correlate of malignant pathology in this cohort.

Table 8 summarizes the association of IRSs with sex and age. Female patients demonstrated significantly higher epithelial IRS values for LIF (Mann–Whitney *p* < 0.0001; rank-biserial r = 0.53) and LIFR (*p* < 0.0001; r = 0.41), indicating sex-related differences in expression of the LIF/LIFR axis. No significant sex differences were observed for IL-6 (*p* = 0.0997) or CXCL9 (*p* = 0.3499).

Age was inversely correlated with LIF (Spearman ρ = −0.277; *p* = 0.0007) and LIFR (ρ = −0.352; *p* < 0.0001), indicating lower epithelial LIF/LIFR expression in older individuals. IL-6 showed no correlation with age (ρ = −0.085; *p* = 0.3067). CXCL9 showed a weak positive trend with age that did not reach statistical significance (ρ = 0.145; *p* = 0.0829). Collectively, these findings suggest that demographic factors, particularly age and sex, are associated with variation in LIF/LIFR expression and should be considered as potential confounders in multivariable analyses.

### 2.7. IRS-IRS and IRS-NBI Associations

#### 2.7.1. IRS–IRS Relationships

Analysis of inter-marker relationships using Spearman’s rank correlation revealed several consistent patterns. IL-6 expression showed modest but significant positive correlations with both LIF (ρ = 0.249, *p* = 0.0026) and LIFR (ρ = 0.261, *p* = 0.0015). The strongest internal association was observed between LIF and LIFR (ρ = 0.526, *p* = 1.13 × 10^−11^), reflecting their shared signaling context within the IL-6/LIF cytokine axis. In contrast, CXCL9 correlated negatively with LIF (ρ = −0.175, *p* = 0.0350) and LIFR (ρ = −0.204, *p* = 0.0137), suggesting divergent regulatory pathways consistent with its immunomodulatory rather than pro-transformational role. Overall, these patterns align with established models of coordinated IL-6/LIF/STAT3 activity.

#### 2.7.2. IRS–NBI Associations

Analysis of IRS values across NBI categories demonstrated distinct and opposing expression patterns among the investigated markers. CXCL9 expression increased with advancing NBI grade and was significantly higher in lesions exhibiting high-risk vascular patterns (Ni ≥ 4), indicating an association between immune-related signaling and advanced microvascular phenotypes. In contrast, both LIF and LIFR expression showed a progressive decline with increasing NBI grade, with the lowest IRS values being observed in lesions classified as high-risk NBI patterns.

Taken together, these findings indicate that high-risk NBI vascular patterns are characterized by concurrent upregulation of immune-associated CXCL9 expression and downregulation of epithelial differentiation-related LIF/LIFR signaling, suggesting parallel processes of inflammatory activation and loss of epithelial differentiation accompanying advanced microvascular remodeling.

IRS values of LIF and LIFR showed clear negative associations with increasing NBI grade, consistent with decreasing epithelial expression in more advanced microvascular patterns (Table S1). In contrast, CXCL9 IRS correlated positively with NBI grade, indicating higher expression in lesions with more suspicious NBI findings. IL-6 did not display a meaningful correlation with NBI grade.

When IRS values were examined across the ordinal Ni classification (types 2–5), coherent trends were observed. LIF decreased across Ni categories (mean IRS: 7.87 in Ni 2, 5.00 in Ni 3, 4.80 in Ni 4, and 2.83 in Ni 5), showing a strong negative Spearman correlation (ρ = −0.561, *p* = 2.24 × 10^−13^). LIFR also demonstrated a significant overall negative association with Ni grade (ρ = −0.481, *p* = 9.39 × 10^−10^; mean IRS: 8.51 in Ni 2, 3.42 in Ni 3, 5.40 in Ni 4, 4.43 in Ni 5), although the mean pattern was not strictly monotonic across all intermediate categories. CXCL9 showed a positive association with increasing Ni grade (ρ = 0.227, *p* = 0.0061; mean IRS: 3.64 in Ni 2, 4.83 in Ni 3, 5.75 in Ni 4, and 4.93 in Ni 5). IL-6 did not vary significantly across Ni categories (ρ = −0.108, *p* = 0.197).

### 2.8. Association Between Tumor Differentiation Grade (G1–G3) and IRS Marker Expression in SCC

Within the SCC subgroup, histological grade (G1–G3) was available for 60 cases (G1 *n* = 10, G2 *n* = 39, G3 *n* = 11). IRS distributions were compared across grades using the Kruskal–Wallis test with Bonferroni-adjusted pairwise Mann–Whitney U tests. Grade was additionally treated as an ordinal variable (1–3) in Spearman correlation analyses.

Overall, no statistically significant differences in IL-6, LIFR, or CXCL9 expression were observed across G1–G3 (all Kruskal–Wallis *p* > 0.5). In contrast, LIF showed a trend toward higher expression in less-differentiated tumors, with median [IQR] increasing from G1 to G3 (Table 9). This was reflected by a borderline overall group difference (Kruskal–Wallis *p* = 0.0515) and a positive ordinal association (Spearman ρ = 0.317, *p* = 0.0137; Benjamini–Hochberg q = 0.0547).

Tumor grade (G1–G3) was not included in the logistic regression for SCC because grading is defined only for SCC cases and is unavailable by design for non-SCC lesions, and it is a postoperative histopathological characteristic rather than a preoperative predictor.

### 2.9. Logistic Regression: Diagnostic Contribution of IHC Beyond NBI

In the univariable logistic regression analyses (outcome: SCC vs. non-SCC, complete-case N = 145), the endoscopic threshold Ni ≥ 4 (Ni4–Ni5) showed the strongest association with carcinoma, conferring an approximately 23-fold increase in the odds of SCC compared with Ni ≤ 3 (OR 22.96, 95% CI 9.64–54.71, *p* = 1.5 × 10^−12^). Older age was also associated with SCC (OR 1.09 per 1-year increase, 95% CI 1.05–1.13, *p* = 1.1 × 10^−5^), and male sex demonstrated higher odds of SCC relative to female sex (OR 5.57, 95% CI 2.49–12.42, *p* = 2.8 × 10^−5^).

Regarding IHC biomarkers, higher LIF and higher LIFR expression were inversely associated with SCC (LIF: OR 0.62 per 1-point IRS increase, 95% CI 0.53–0.73, *p* = 4.8 × 10^−9^; LIFR: OR 0.68, 95% CI 0.60–0.78, *p* = 1.7 × 10^−8^), suggesting that greater expression of these markers is more frequently observed in non-malignant lesions. In contrast, CXCL9 was positively associated with SCC (OR 1.19 per IRS point, 95% CI 1.05–1.34, *p* = 0.005), while IL-6 did not show a significant univariable association (OR 0.95, 95% CI 0.86–1.06, *p* = 0.365). Collectively, these unadjusted results indicate that endoscopic risk stratification (Ni ≥ 4) is the dominant single predictor of SCC, with age and sex contributing additional baseline-risk signals, and with selected biomarkers (particularly CXCL9 and inversely LIF/LIFR) showing measurable univariable associations that warrant evaluation in multivariable models (Table 10).

In the multivariable logistic regression model (outcome: SCC vs. non-SCC; complete-case N = 145), the endoscopic threshold Ni ≥ 4 (Ni4–Ni5) remained an independent predictor of carcinoma after adjustment for age, sex, and IHC biomarker expression (IRS) for IL-6, LIF, LIFR, and CXCL9. Specifically, Ni ≥ 4 was associated with an almost nine-fold increase in the odds of SCC compared with Ni ≤ 3 (adjusted OR 8.90, 95% CI 2.97–26.62, *p* < 0.001), confirming that a high-risk NBI pattern retains strong predictive value even when demographic and tissue biomarker variables are considered.

Among the biomarkers, higher LIF and LIFR expression were independently associated with lower odds of SCC (LIF: adjusted OR 0.73 per 1-point IRS increase, 95% CI 0.61–0.88, *p* = 0.001; LIFR: adjusted OR 0.78, 95% CI 0.65–0.93, *p* = 0.006), indicating an inverse relationship in which greater expression is more frequently observed in non-malignant lesions. In contrast, IL-6 and CXCL9 were not independently associated with SCC in the adjusted model (IL-6: adjusted OR 1.11, *p* = 0.302; CXCL9: adjusted OR 1.09, *p* = 0.349). Age showed only a non-significant trend toward higher SCC odds (adjusted OR 1.05 per year, *p* = 0.075), and male sex was not an independent predictor after adjustment (adjusted OR 1.88, *p* = 0.382). Overall, the model demonstrated excellent discriminatory performance (AUC = 0.943), indicating strong overall discrimination, with NBI remaining the dominant contributor and biomarkers providing secondary refinement to differentiate SCC from non-SCC lesions in this cohort (Table 11). Overall model fit was supported by likelihood-based diagnostics, indicating adequate robustness and discrimination of the final multivariable model.

The ROC analysis comparing individual predictors for SCC (complete-case cohort, N = 145) demonstrated that the binary endoscopic threshold NBI Ni ≥ 4 provided the best single-variable discrimination between SCC and non-SCC lesions (AUC = 0.83). Age showed moderate discriminatory ability (AUC = 0.73), indicating increasing SCC probability with older age, while male sex provided only modest discrimination (AUC = 0.68).

**Figure 2:**
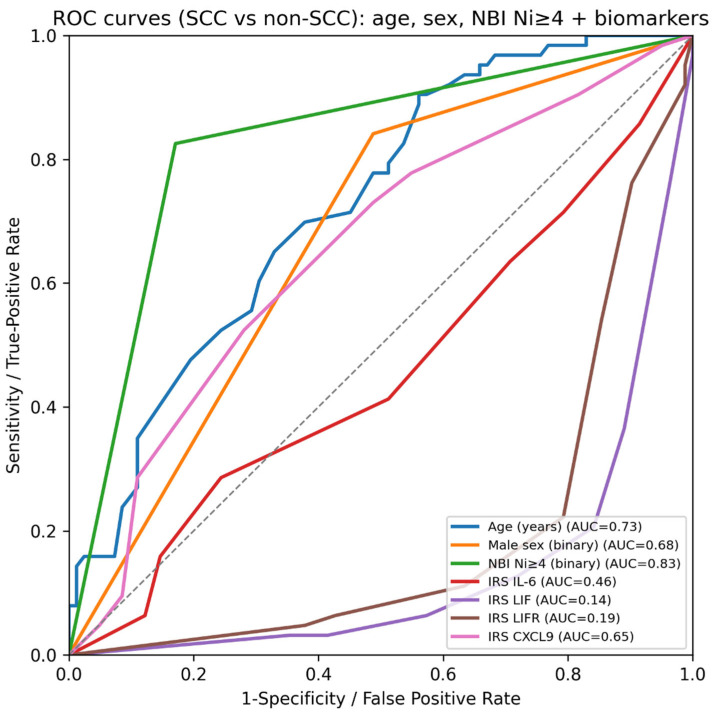
Receiver operating characteristic (ROC) curves for discrimination of squamous cell carcinoma (SCC) versus non-SCC lesions using individual predictors. AUC, area under the curve; IRS, immunoreactive score; NBI, Narrow-Band Imaging.

Among the IHC biomarkers, CXCL9 achieved moderate performance (AUC = 0.65), whereas IL-6 showed poor discrimination (AUC = 0.46), consistent with limited diagnostic utility when used alone. LIF (AUC = 0.14) and LIFR (AUC = 0.19) displayed AUC values well below 0.50, reflecting an inverse relationship with SCC; in other words, higher LIF/LIFR expression was more typical of non-malignant lesions, and reversing the direction of these markers would correspond to AUC values of approximately 0.86 and 0.81, respectively. Overall, this ROC comparison indicates that endoscopic stratification (Ni ≥ 4) outperforms individual biomarkers and demographic variables, while selected tissue markers, particularly CXCL9, and inversely LIF/LIFR may add complementary information when integrated into multivariable prediction models (Figure 2).

### 2.10. ROC Analysis (SCC vs. Non-SCC)

ROC curves were constructed to assess the diagnostic performance of demographic variables, NBI classification, and immunohistochemical markers in differentiating squamous cell carcinoma (SCC) from non-SCC lesions in the complete-case cohort (N = 145). IL-6 showed near-chance discrimination (AUC = 0.46), whereas LIF and LIFR exhibited inverse discrimination (AUC = 0.14 and 0.19, respectively), indicating that lower expression was associated with higher SCC risk (Table 12). CXCL9 demonstrated the best discriminatory ability among the IHC markers (AUC = 0.65), consistent with modest performance when used as a single predictor. In contrast, NBI provided substantially stronger diagnostic accuracy: the binary high-risk classification Ni ≥ 4 (Ni4–Ni5) achieved an AUC of 0.83, outperforming both age (AUC = 0.73) and sex (AUC = 0.68). Overall, these results indicate that endoscopically assessed microvascular abnormalities (captured by the Ni ≥ 4 threshold) offer markedly greater diagnostic value for SCC than individual tissue cytokine markers, while CXCL9 and the inverse signal of LIF/LIFR may provide complementary information in multivariable models. Notably, AUC values below 0.50 for LIF and LIFR reflect the opposite direction of association; reversing the predictor scale corresponds approximately to AUC_rev = 1 − AUC (i.e., ~0.86 for LIF and ~0.81 for LIFR).

## 3. Discussion

This study combines endoscopic assessment of microvascular patterns with tissue-level immunohistochemical analysis to explore potential biological associations between cytokine signaling and NBI-visible vascular remodeling. Although we constructed an exploratory multivariable model to assess independent associations with SCC, the primary aim of this study was mechanistic interpretation rather than development of a clinical decision tool.

We found that expression of LIF/LIFR is higher in women; however, the present study was not designed to investigate underlying mechanisms, this finding may reflect sex-related biological differences, potentially involving hormonal regulation or immune modulation, and should therefore be regarded as hypothesis-generating. Furthermore, exploratory analyses did not identify statistically significant interaction effects between sex and the main predictors. The central finding is that NBI microvascular grading (Ni classification) remained the most accurate discriminator of squamous cell carcinoma, consistent with the fact that endoscopic microvasculature represents a phenotype resulting from epithelial–stromal crosstalk rather than epithelial signaling alone.

In our cohort, the Ni ≥ 4 cut-off showed balanced sensitivity and specificity for SCC, supporting its practical value for endoscopic risk stratification. Previous studies evaluating the Ni classification in laryngeal lesions have reported sensitivity values ranging from approximately 75–90% and specificity between 70 and 85% for the detection of high-grade dysplasia or squamous cell carcinoma. The diagnostic performance observed in the present cohort (sensitivity 82.5%, specificity 82.9%) is consistent with these findings, supporting the robustness and reproducibility of the Ni ≥ 4 threshold across different clinical settings [26,27].

The decrease in LIF and LIFR expression with increasing histopathological severity is consistent with recent observations in oral SCC reported by Yakin et al. (2025), who noted higher LIF expression in normal mucosa with lower expression in tumors and emphasized the relationship between reduced differentiation-associated signaling and malignant phenotypes [20]. This interpretation aligns with the broader concept of cancer hallmarks related to phenotypic plasticity and altered differentiation programs [28]. In our cohort, non-dysplastic epithelium exhibited the highest LIF IRS with a marked decline in SCC, supporting the view that LIF/LIFR signaling in squamous epithelium may be linked to lineage stability and regulated proliferation.

At the same time, LIF/LIFR signaling is widely recognized as context-dependent and may exert tumor-suppressive or pro-tumorigenic effects depending on tissue and microenvironment [16,29] Christianson et al. (2021) emphasized that LIF/LIFR biology is tightly coupled to inflammation, stromal signaling, and immune evasion, while Španko et al. (2021) argued that IL-6 family signaling often operates at the level of the entire tissue ecosystem rather than being captured by single-compartment tumor-cell biomarkers [12,17]. In our dataset, LIF and LIFR showed a strong positive correlation, consistent with their shared signaling pathway. In contrast, their weaker associations with IL-6 suggest that these cytokines may be regulated through partially independent mechanisms and possibly originate from different cellular compartments. This pattern supports coordinated pathway-level regulation rather than isolated changes in single epithelial markers.

Although IL-6 is a well-established driver of tumor-promoting inflammation and STAT3 activation, its epithelial IRS did not differ significantly across WHO 2017 categories in this cohort. This does not negate IL-6’s relevance; rather, it is compatible with compartment-specific biology highlighted by Orange et al. (2023), who described IL-6 classic signaling as relatively homeostatic while trans-signaling predominates in tumor microenvironments and is strongly pro-inflammatory and pro-tumorigenic [14]. In this framework, epithelial IL-6 IRS may fail to capture stromal/immune IL-6 sources, IL-6R shedding, and endothelial responses that ultimately drive angiogenic remodeling. Thus, high-risk NBI patterns (including the Ni ≥ 4 high-risk threshold) may represent downstream vascular consequences of IL-6-dependent microenvironmental trans-signaling and related stromal pathways, rather than epithelial IL-6 levels detectable by standard IHC. Accordingly, the absence of significant epithelial IRS differences does not preclude an important functional role of IL-6 in malignant progression. Thus, in this dataset, IL-6 appears biologically relevant at the microenvironmental level but not informative as an epithelial IHC marker.

CXCL9 displayed a different signature. Although post hoc differences between benign and premalignant lesions were limited, CXCL9 increased with higher Ni grades and correlated positively with NBI severity, suggesting that it reflects immune–vascular activation and inflammatory remodeling rather than epithelial atypia alone. This interpretation aligns with the dual role of CXCL9 described in the literature: Tokunaga et al. (2018) highlighted its potential to promote anti-tumor immunity via T-cell recruitment, whereas Nagarsheth et al. (2017) discussed its relevance for immunotherapy responsiveness and frequent epigenetic suppression in tumors [23,24]. In our cohort, the association between CXCL9 and high-risk NBI patterns suggests that microvascular phenotypes may be associated with lesions not yet histologically invasive.

A key strength of this study is the parallel evaluation of cytokine marker expression with NBI microvascular phenotypes. LIF and LIFR decreased in high-risk NBI categories, consistent with reduced differentiation-associated signaling, while CXCL9 increased in more suspicious vascular patterns, consistent with immune–vascular activation. IL-6 showed weak epithelial IRS signals despite its established biological importance, likely reflecting dominance of microenvironmental trans-signaling. From a biological perspective, NBI-visible vascular alterations represent the phenotypic endpoint of angiogenic remodeling driven by cytokine signaling, immune activation, and extracellular matrix reorganization. The observed inverse association of LIF and LIFR with high-risk NBI patterns likely reflects loss of differentiation-associated epithelial signaling, whereas the positive association of CXCL9 with advanced NBI grades may indicate immune vascular activation within the tumor microenvironment. LIF/LIFR signaling intersects with pathways that regulate extracellular matrix (ECM) remodeling and epithelial–mesenchymal plasticity—processes tightly coupled to angiogenesis. Although LIF is frequently discussed as a differentiation-associated cytokine in squamous epithelia, LIFR functions as a signaling platform capable of engaging JAK/STAT, ERK/MAPK and PI3K/Akt cascades, which influence EMT programs, matrix turnover, and pro-angiogenic mediator release. Importantly, Zhou et al. (2022) demonstrated that LIFR activation within an ILEI–LIFR complex can promote EMT through Akt and ERK phosphorylation, linking LIFR signaling to EMT/ECM-associated remodeling [22]. This provides biological plausibility that the vascular phenotypes visualized by NBI (high-risk Ni patterns) may reflect integrated remodeling of the epithelial–stromal unit, including ECM dynamics, rather than epithelial cytokine abundance alone.

At a conceptual level, and assuming future availability of preoperative or rapid assays, NBI-based microvascular grading remains the most actionable tool.

In our cohort, the Ni ≥ 4 threshold provided balanced sensitivity and specificity for squamous cell carcinoma, supporting its use to prioritize oncologic work-up and to plan an adequate extent of transoral laser microsurgery (TOLMS) when malignancy is suspected. Although tissue IRS markers are not currently available as intraoperative or preoperative tests, the inverse association of LIF and LIFR with SCC suggests that reduced epithelial LIF/LIFR signaling may identify lesions with a more ‘plastic’ biology that merits heightened vigilance. In practice, if validated in prospective settings and translated into rapid or minimally invasive assays, LIF/LIFR profiling could serve as an adjunct in borderline endoscopic scenarios (e.g., equivocal Ni patterns) to support decisions such as more-extensive mapping biopsies, wider resection margins during partial procedures, or intensified postoperative surveillance. At present, these applications should be regarded as hypothesis-generating rather than practice-changing, given the cross-sectional design and tissue-based measurement of biomarkers.

This study has several limitations. First, it is cross-sectional, so we cannot infer temporal “malignant transformation” trajectories. Second, while our IHC approach quantified epithelial IRS, it did not separately quantify stromal, endothelial or immune compartments, which are central to IL-6 trans-signaling and vascular remodeling. Third, we did not directly quantify ECM remodeling (e.g., collagen composition, MMP activity, or stromal activation markers), which likely mediates the link between cytokine signaling, EMT-like plasticity, and angiogenic phenotypes observed in NBI. This limitation is particularly relevant for IL-6, the pro-angiogenic and tumor-promoting effects of which in LSCC are largely mediated through microenvironmental trans-signaling rather than epithelial cytokine abundance.

Future work should use multiplex/spatial methods (multiplex IHC, spatial transcriptomics, or compartment-specific scoring) to disentangle epithelial vs. stromal contributions, explicitly capture IL-6 trans-signaling components, and map immune–vascular niches underlying high-risk NBI phenotypes. Longitudinal studies would be valuable to clarify whether early shifts in LIF/LIFR or CXCL9 predict subsequent histological progression and recurrence.

Potential translational applications would require future development of preoperative or rapid tissue-based assays, such as targeted immunostaining on biopsy specimens, brush cytology, or spatial molecular profiling, which were beyond the scope of the present study.

Therefore, all proposed clinical applications should be regarded as exploratory and hypothesis-generating rather than practice-changing at this stage.

Because biomarker assessment was tissue-based and performed postoperatively, we cannot infer how LIF/LIFR measurement would alter intraoperative decision-making; prospective studies incorporating preoperative sampling or rapid assays are required. Future studies in larger cohorts should specifically evaluate biomarker expression in false-positive and false-negative NBI cases to determine whether molecular profiling provides incremental diagnostic or prognostic value in equivocal endoscopic scenarios.

## 4. Materials and Methods

### 4.1. Study Design and Patient Cohort

A total of 145 patients with vocal fold lesions, qualified for surgical intervention, were prospectively enrolled between November 2020 and June 2023 at the Tertiary Department of Otolaryngology, Medical University of Łódź, Poland. The sample size was determined by patient availability rather than by a priori power calculation.

The study was conducted in accordance with the Declaration of Helsinki. Ethical approval was obtained from the Bioethics Committee of the Medical University of Łódź (decision No. RNN/257/20/KE), and written informed consent was secured from all participants. Postoperative follow-up adhered to the Polish ENT Society Board consensus [30].

Inclusion criteria were as follows: (1) age ≥ 18 years; (2) clinical diagnosis of vocal fold lesion requiring surgical biopsy or excision; (3) availability of high-quality preoperative endoscopic evaluation in both white-light imaging (WLI) and Narrow-Band Imaging. Exclusion criteria included: (1) history of other head and neck malignancies; (2) prior head and neck cancer treatment; (3) active infectious disease at the time of surgery; (4) systemic immunosuppression; (5) poor-quality endoscopic imaging; (6) lack of written informed consent. Medical history and clinicopathological data were recorded for all participants. Tumor staging and classification were performed according to TNM/UICC guidelines [31]

### 4.2. Endoscopic Assessment

All patients underwent transnasal, flexible video-laryngoscopy (ENF-VH2, Olympus Corp., Tokyo, Japan) on the day of surgery in a seated position. Examinations were performed by an experienced otolaryngologist (WP). Topical lidocaine spray was administered in selected cases when a strong gag reflex was present. Each examination commenced with WLI, followed by NBI to enhance the detection of mucosal microvascular patterns. Still images and representative video sequences were archived for each patient.

#### Narrow-Band Imaging (NBI) Evaluation

Two observers independently assessed NBI images (MB, WP); disagreements were resolved by consensus.

For NBI assessment, we used Ni et al.’s (2011) classification system [9]. Vascular architecture was scored from Type 1 to Type 5.

In this cohort, no lesions were classified as Ni Type I; therefore, subsequent analyses were restricted to lesions graded Ni Types II–V.

For the main statistical analyses and clinical risk stratification, Ni grades were dichotomized using a single pre-specified threshold: low-risk Ni types 1–3 versus high-risk Ni types 4–5 (operationally, Ni II–III versus Ni IV–V in this cohort). This dichotomization was used in contingency analyses, ROC analyses, and logistic regression modeling for SCC [8,9,32].

Inter- and intraobserver agreement for NBI scoring (1–5 ordinal scale) was assessed using weighted Cohen’s kappa with quadratic weights, appropriate for ordinal categorical variables. For interobserver reliability, NBI ratings assigned independently by two observers (MB and WP) for all 145 cases were compared. For intraobserver reliability, one observer (MB) repeated the evaluation four weeks after the initial scoring under identical blinded conditions. Weighted κ values were interpreted according to the Landis and Koch criteria.

### 4.3. Surgical Procedures

Surgical interventions were performed according to clinical indication and included transoral laser microsurgery and total laryngectomy. Resected specimens were processed according to routine pathological protocols.

### 4.4. Immunohistochemistry (IHC)

IHC was performed to assess expression of IL-6, LIF, LIFR, and CXCL9. Primary antibodies used in research: -IL-6: Affinity Biosciences, Cat. #DF6087, RRID:AB_2838055. -LIF: Affinity Biosciences, Cat. #DF13730, RRID:AB_2846749. -CXCL9: Affinity Biosciences, Cat. #DF9920, RRID:AB_2843114. -LIFR: Affinity Biosciences, Cat. #DF13129, RRID:AB_2846089.

#### Tissue Processing and Staining Protocol

Laryngeal tissues were fixed in 4% paraformaldehyde for 8 h at room temperature, embedded in paraffin and sectioned at 3 µm. Sections were dried at 60 °C for 1 h. Sections were deparaffinized and rehydrated using standard protocols. For IHC, antigen retrieval was performed by heating slides at 97 °C for 20 min in Dako Target Retrieval Solution (Agilent Technologies, Santa Clara, CA, USA). After washing in Tris-buffered saline (TBS), slides were incubated with the respective primary mouse monoclonal antibodies at a 1:500 dilution for 20 h at room temperature, following the manufacturer’s instructions and in-house optimization. Detection was performed using the EnVision system (Dako; Agilent Technologies) with horseradish peroxidase and visualization with 3,3′-diaminobenzidine (DAB).

Slides were evaluated independently by two investigators using light microscopy at ×200 and ×400 magnification. Protein expression was scored using the immunoreactive score (IRS) [33], which multiplies staining intensity (0–3) by the percentage of positive cells (0–4) to yield scores ranging from 0 to 12. Based on IRS values, protein expression was categorized as follows:

0–1: negative;

2–3: positive—weak expression;

4–8: positive—mild expression;

9–12: positive—strong expression.

Immunoreactivity was evaluated exclusively in the epithelial compartment. Only lesional epithelial cells were considered for IRS scoring, whereas stromal immune cells, endothelial cells and other non-epithelial elements were not quantified. Hot spots of the most intense staining were identified at low magnification, and IRS was then assigned at higher magnification by averaging the staining intensity and percentage of positive cells across the entire lesional epithelium. After incubation with the primary antibodies, sections were counterstained with hematoxylin to visualize cell nuclei. Positive control tissues were selected according to the manufacturer’s recommendations for each antibody, and negative controls were processed identically with omission of the primary antibody.

This classification was implemented programmatically using a standardized logical formula in the data sheet to ensure consistent interpretation across all samples.

### 4.5. Data Management and Statistical Analysis

For statistical analyses, histopathology was reported according to the WHO 2017 classification and additionally grouped as non-dysplastic lesions, low-grade dysplasia (LGD), high-grade dysplasia (HGD), and invasive squamous cell carcinoma (SCC). The primary endpoint for predictive modeling and diagnostic accuracy analyses was SCC (yes/no); all non-SCC categories served as the reference group.

NBI Ni grades were analyzed descriptively across histopathological categories and, for the main inferential analyses, dichotomized into low-risk (Ni types 1–3) and high-risk (Ni types 4–5) [9]. In this cohort, this corresponded to Ni 2–3 versus Ni 4–5.

Univariable logistic regression models were fitted separately for each candidate predictor. Variables showing at least a weak association in univariable analyses (*p* < 0.10) were considered for inclusion in multivariable logistic regression. The multivariable model included the high-risk NBI threshold (Ni 4–5 vs. Ni 2–3), age (years), sex, and epithelial IHC IRSs for IL-6, LIF, LIFR, and CXCL9, entered simultaneously using the enter method. Collinearity between predictors was assessed using variance inflation factors, and no problematic collinearity was observed. Multivariable models were fitted using complete-case analysis for all included predictors. Multicollinearity among predictors was assessed using variance inflation factors, and no problematic collinearity was observed.

To account for multiple testing, *p*-values were adjusted using the Benjamini–Hochberg false-discovery rate (FDR) procedure. FDR correction was applied separately within pre-specified families of tests, namely: comparisons of IRS values across histopathological categories, correlations between IRS and NBI grade, and associations of IRS with sex and age. An FDR-adjusted q-value below 0.10 was considered statistically significant. For regression models, two-sided *p*-values < 0.05 were considered statistically significant. NBI findings and tissue biomarkers were analyzed as independent variables; no composite or integrated diagnostic model combining NBI and biomarker data was constructed.

Cases with missing IRS or NBI values were excluded pairwise from the respective analyses, and no imputation of missing data was performed.

Continuous IRS variables were tested for normality using the Shapiro–Wilk test. Depending on distribution, group comparisons were performed using Student’s *t*-test or Mann–Whitney U test (two groups) and one-way ANOVA with Tukey’s test or Kruskal–Wallis with Dunn–Bonferroni correction (≥3 groups). Effect sizes were reported as Cohen’s d, η^2^, or rank-based r/ε^2^. Associations between categorical variables were evaluated using the Chi-square test or Fisher’s exact test, with corresponding odds ratios (ORs) and 95% CIs. Correlations between markers and NBI grades were assessed using Spearman’s rho. Diagnostic performance for identifying high-grade dysplasia or carcinoma was analyzed using ROC curves (AUC, 95% CI), both for individual predictors and for multivariable model-derived predicted probabilities. Inter- and intraobserver agreement for NBI scoring was evaluated using Cohen’s kappa (unweighted and weighted), and agreement for continuous IRS values using the intraclass correlation coefficient (ICC). Multiple comparisons were controlled using the Benjamini–Hochberg FDR procedure. Statistical significance was set at *p* < 0.05. Statistical analyses were performed in the Statistica 13.1 software and GraphPad Prism 10.

### 4.6. Data Availability

The de-identified individual-participant data that underlie the results reported in this article are available on reasonable request from the corresponding author, subject to institutional approvals and data-sharing agreements.

## 5. Conclusions

In vocal fold lesions, NBI microvascular grading using the Ni classification—particularly the Ni ≥ 4 threshold—showed strong discrimination for squamous cell carcinoma and outperformed epithelial IRS markers. LIF and LIFR were inversely associated with SCC and high-risk NBI patterns, consistent with loss of differentiation-linked signaling, whereas CXCL9 tracked high-risk vascular phenotypes consistent with immune–vascular activation. Epithelial IL-6 IRS did not reliably reflect histological severity, supporting the concept that IL-6-driven angiogenic effects are mainly mediated by microenvironmental trans-signaling rather than epithelial IL-6 abundance. Overall, NBI appears to capture the integrated downstream phenotype of cytokine-, immune-, and ECM-associated remodeling, offering a clinically robust, real-time surrogate of microenvironmental activity.

## Figures and Tables

**Table 1:** Clinicopathological characteristics of the study cohort.

Variable	*n* (%)
Total	145 (100.0)
Sex	
Female	52 (35.9)
Male	93 (64.1)
Age (median)	68 (35–89)
Clinical presentation	
Tumor	71 (49.0)
Leukoplakia	29 (20.0)
Benign lesions (polyps, Reinke’s edema)	45 (31)
NBI (Ni et al.) [9]	
Type I	0
Type II	67 (46.2)
Type III	12 (8.3)
Type IV	20 (13.8)
Type V	46 (31.7)
Type of procedure	
TOLMS	127 (87.6)
Total laryngectomy	18 (12.4)
Histopathology	
Non-dysplastic epithelium	59 (40.7)
Low-grade dysplasia (LGD)	11 (7.6)
High-grade dysplasia (HGD)	12 (8.3)
Squamous cell carcinoma (SCC)	63 (43.4)
SCC grade (*n* = 63)	
G1	11 (17.5 of SCC)
G2	40 (63.5 of SCC)
G3	12 (19.0 of SCC)
T status (SCC; *n* = 63)	
T1	34 (54.0 of SCC)
T2	8 (12.7 of SCC)
T3	10 (15.9 of SCC)
T4	11 (17.5 of SCC)
N status (SCC; *n* = 63)	
N0	49
N1	8
N2	6
N3	0

**Table 2:** Clinical presentation groups versus WHO 2017 histopathology (N = 145). Values are n (row %), showing the distribution of WHO 2017 histopathology within each clinical presentation group.

Clinical Presentation Group	Non-Dysplastic	LGD	HGD	SCC	Row Total
Benign (polyp/Reinke/cyst)	41 (91.1%)	3 (6.7%)	1 (2.2%)	0 (0.0%)	45
Premalignant (leukoplakia)	4 (13.8%)	2 (6.9%)	8 (27.6%)	15 (51.7%)	29
Exophytic tumor	14 (19.7%)	6 (8.5%)	3 (4.2%)	48 (67.6%)	71
Column total	59	11	12	63	145

The clinical presentation groups are descriptive and may not correspond directly to histopathological severity; all inferential analyses were based on WHO 2017 histopathology.

**Table 3:** Association between NBI (Ni classification) and histopathology (WHO 2017).

NBI Ni	Non-Dysplastic Epithelium (%)	Low-Grade Dysplasia (%)	High-Grade Dysplasia (%)	Squamous Cell Carcinoma (%)	Column Total
Ni 2	56 (94.9)	5 (45.5)	0 (0.0)	6 (9.5)	67
Ni 3	1 (1.7)	0 (0.0)	6 (50.0)	5 (7.9)	12
Ni 4	1 (1.7)	4 (36.4)	4 (33.3)	11 (17.5)	20
Ni 5	1 (1.7)	2 (18.2)	2 (16.7)	41 (65.1)	46
Row total	59	11	12	63	145

**Table 4:** Diagnostic performance of the Ni ≥ 4 threshold for SCC detection (combined contingency components and accuracy measures).

Section	Parameter	Definition	Value
Contingency components	True positives (TP)	Ni ≥ 4 and SCC	52
	False positives (FP)	Ni ≥ 4 and non-SCC	14
	False negatives (FN)	Ni ≤ 3 and SCC	11
	True negatives (TN)	Ni ≤ 3 and non-SCC	68
	Total SCC		63
	Total non-SCC		82
	Total (N)		145
Diagnostic accuracy	Sensitivity	TP/(TP + FN)	52/63 (82.5%)
	Specificity	TN/(TN + FP)	68/82 (82.9%)
	PPV	TP/(TP + FP)	52/66 (78.8%)
	NPV	TN/(TN + FN)	68/79 (86.1%)
	Accuracy	(TP + TN)/N	120/145 (82.8%)
	LR+	Sensitivity/(1 − Specificity)	4.83
	LR−	(1 − Sensitivity)/Specificity	0.21

Abbreviations: SCC, squamous cell carcinoma; PPV, positive predictive value; NPV, negative predictive value; LR+, positive likelihood ratio; LR−, negative likelihood ratio.

**Table 5:** Odds ratios (ORs) for squamous cell carcinoma (SCC) by NBI Ni category (reference: Ni2).

Comparison	SCC/N (%)	Non-SCC (*n*)	OR	95% CI
Ni3 vs. Ni2	5/12 (41.7%)	7	7.26	1.75–30.09
Ni4 vs. Ni2	11/20 (55.0%)	9	12.43	3.68–41.93
Ni5 vs. Ni2	41/46 (89.1%)	5	83.37	23.86–291.30
Primary cut-off (Ni ≥ 4 vs. Ni ≤ 3)				
Ni ≥ 4 vs. Ni ≤ 3	52/66 (78.8%) vs. 11/79 (13.9%)	14 vs. 68	22.96	9.64–54.71

Abbreviations: SCC, squamous cell carcinoma; OR, odds ratio; CI, confidence interval. Percentages in the ‘SCC/N (%)’ column refer to the proportion of SCC within each Ni category.

**Table 6:** IRS expression across histopathological groups (WHO 2017): Kruskal–Wallis and post hoc Mann–Whitney with Bonferroni correction. Values are median [IQR]. Post hoc *p*-values are Bonferroni-adjusted (*p*_adj).

Marker	Benign	LGD	HGD	SCC	KW *p*	ε^2^	Benign vs. LGD	Benign vs. HGD	Benign vs. SCC	LGD vs. HGD	LGD vs. SCC	HGD vs. SCC
IL-6 (IRS)	6.0 [4.0–8.0]	4.0 [2.0–6.0]	4.0 [2.0–6.0]	4.0 [2.0–8.0]	0.4412	0.0	1	1	1	1	1	1
LIF (IRS)	8.0 [6.0–12.0]	6.0 [4.5–10.5]	4.0 [2.0–6.8]	2.0 [2.0–3.0]	5.5 × 10^−14^	0.438	1	0.0322	1.2 × 10^−14^	1	0.0156	0.1144
LIFR (IRS)	8.0 [7.0–12.0]	9.0 [4.5–12.0]	3.5 [2.0–6.0]	4.0 [3.0–4.0]	4.4 × 10^−12^	0.375	1	0.0006	1.9 × 10^−12^	0.4449	0.0642	1
CXCL9 (IRS)	2.0 [2.0–5.0]	4.0 [2.0–6.0]	4.0 [3.5–6.0]	6.0 [3.0–8.0]	0.0078	0.063	1	1	0.0040	1	1	1

Group sizes: IL-6 (IRS): Benign *n* = 59, LGD *n* = 11, HGD *n* = 12, SCC *n* = 63; LIF (IRS): Benign *n* = 59, LGD *n* = 11, HGD *n* = 12, SCC *n* = 63; LIFR (IRS): Benign *n* = 59, LGD *n* = 11, HGD *n* = 12, SCC *n* = 63; CXCL9 (IRS): Benign *n* = 59, LGD *n* = 11, HGD *n* = 12, SCC *n* = 63. Abbreviations: IRS, immunoreactive score; IQR, interquartile range; LGD, low-grade dysplasia; HGD, high-grade dysplasia; SCC, squamous cell carcinoma.

**Table 7:** Age distribution across clinicopathological categories.

Histopathology	Median	Mean	SD	Min–Max	N
Benign	59.0	58.47	12.29	35–83	59
LGD	70.0	67.60	9.40	44–76	11
HGD	71.0	68.92	9.01	50–79	12
SCC	72.0	70.67	8.58	46–89	63
Non-SCC	65.5	61.28	12.24	35–83	82
SCC	72.0	70.67	8.58	46–89	63

Kruskal–Wallis test across categories: *p* = 4.6 × 10^−7^ Abbreviations: LGD, low-grade dysplasia; HGD, high-grade dysplasia; SCC, squamous cell carcinoma; SD, standard deviation. Mann–Whitney U test (two-sided): *p* = 6.0 × 10^−6^; rank-biserial r = 0.44. Abbreviations: SCC, squamous cell carcinoma; SD, standard deviation.

**Table 8:** Analysis of IRS vs. sex (male vs. female) and IRS vs. age.

Marker	Median IRS (Male)	Median IRS (Female)	Sex Difference (Mann–Whitney *p*)	Effect Size (Rank-Biserial r)	Age Correlation (Spearman ρ)	Age *p*-Value
IL-6	4.0	5.0	0.0997	0.163 (small)	−0.085	0.3067
LIF	3.0	8.0	<0.0001	0.534 (large)	−0.277	0.0007
LIFR	4.0	8.0	<0.0001	0.413 (moderate)	−0.352	<0.0001
CXCL9	4.0	4.0	0.3499	−0.093 (negligible)	0.145	0.0829 (trend)

**Table 9:** IRS marker expression by tumor grade in SCC (N = 60). Values are median [IQR]. Post hoc *p*-values are shown as raw *p* → Bonferroni-adjusted *p* (*p*_adj).

Marker	G1	G2	G3	KW *p*	ε^2^ (KW)	G1 vs. G2	G1 vs. G3	G2 vs. G3	Spearman ρ	Spearman *p*	FDR q
IL-6 (IRS)	3.5 [2.0–7.5]	4.0 [2.5–8.0]	4.0 [3.0–7.0]	0.8780	0.000	0.7070 → 1.0000	0.6428 → 1.0000	0.8218 → 1.0000	0.065	0.6224	0.6224
LIF (IRS)	2.0 [1.0–2.0]	2.0 [2.0–3.0]	3.0 [2.0–4.0]	0.0515	0.069	0.1164 → 0.3493	0.0193 → 0.0578	0.1410 → 0.4231	0.317	0.0137	0.0547
LIFR (IRS)	3.0 [3.0–3.8]	4.0 [2.0–4.0]	4.0 [3.5–4.0]	0.6350	0.000	0.8098 → 1.0000	0.2723 → 0.8170	0.4768 → 1.0000	0.119	0.3660	0.6224
CXCL9 (IRS)	6.0 [4.5–8.0]	4.0 [3.5–6.0]	4.0 [2.0–8.5]	0.5210	0.000	0.2237 → 0.6712	0.6940 → 1.0000	0.8303 → 1.0000	−0.083	0.5263	0.6224

Abbreviations: SCC, squamous cell carcinoma; IRS, immunoreactive score; IQR, interquartile range; KW, Kruskal–Wallis; FDR, false discovery rate (Benjamini–Hochberg).

**Table 10:** Univariable logistic regression for SCC (yes/no). Univariable logistic regression models were fitted separately for each predictor. Outcome: SCC (yes/no). Complete-case dataset consistent with the multivariable model; N = 145. Continuous variables are reported per 1-unit increase (years or IRS points).

Predictor	Unadjusted OR	95% CI	*p*-Value
Ni ≥ 4 (Ni4–Ni5) vs. Ni ≤ 3 (Ni2–Ni3)	22.96	9.64–54.71	1.5 × 10^−12^
Age (per 1 year)	1.09	1.05–1.13	1.1 × 10^−5^
Male sex (vs female)	5.57	2.49–12.42	2.8 × 10^−5^
IL-6 (IRS, per 1-point increase)	0.95	0.86–1.06	0.365
LIF (IRS, per 1-point increase)	0.62	0.53–0.73	4.8 × 10^−9^
LIFR (IRS, per 1-point increase)	0.68	0.60–0.78	1.7 × 10^−8^
CXCL9 (IRS, per 1-point increase)	1.19	1.05–1.34	0.005

Abbreviations: SCC, squamous cell carcinoma; OR, odds ratio; CI, confidence interval; IRS, immunoreactive score.

**Table 11:** Multivariable logistic regression model for squamous cell carcinoma (SCC). Outcome: SCC (yes/no). Predictors: Ni ≥ 4, age (years), sex, and IHC IRSs for IL-6, LIF, LIFR, and CXCL9. Complete-case analysis; N = 145. Model discrimination: AUC = 0.943.

Predictor	Adjusted OR	95% CI	*p*-value
Ni ≥ 4 (Ni4–Ni5) vs. Ni ≤ 3 (Ni2–Ni3)	8.90	2.97–26.62	<0.001
Age (per 1 year)	1.05	0.99–1.12	0.075
Male sex (vs female)	1.88	0.46–7.77	0.382
IL-6 (IRS, per 1-point increase)	1.11	0.91–1.36	0.302
LIF (IRS, per 1-point increase)	0.73	0.61–0.88	0.001
LIFR (IRS, per 1-point increase)	0.78	0.65–0.93	0.006
CXCL9 (IRS, per 1-point increase)	1.09	0.91–1.32	0.349

Abbreviations: OR, odds ratio; CI, confidence interval; AUC, area under the ROC curve; IRS, immunoreactive score. Adjusted ORs for biomarkers represent the change in odds of SCC per 1-point increase in IRS.

**Table 12:** ROC analysis of individual predictors for SCC (N = 145).

Predictor	AUC	Interpretation
Age (years)	0.73	Moderate discrimination
Male sex (binary)	0.68	Modest discrimination
NBI Ni ≥ 4 (binary)	0.83	Good discrimination
IL-6 (IRS)	0.46	No diagnostic value
LIF (IRS)	0.14	Inverse discrimination (AUC_rev ≈ 0.86)
LIFR (IRS)	0.19	Inverse discrimination (AUC_rev ≈ 0.81)
CXCL9 (IRS)	0.65	Modest discrimination

Abbreviations: AUC_rev indicates the AUC after reversing the direction of the predictor (AUC_rev = 1 − AUC), used to interpret inverse discrimination (AUC < 0.50).

## Data Availability

The data presented in this study are available on request from the corresponding author due to privacy and legal reasons.

## Supplementary Materials

The following supporting information can be downloaded at: https://www.mdpi.com/article/10.3390/ijms27041923/s1.
