# Supplementary material for: Tissue IL-6/LIF/LIFR and CXCL9 Expression Correlates with High-Risk NBI Patterns and Squamous Cell Carcinoma in Vocal Fold Lesions
Source: Int J Mol Sci. 2026 Feb 17;27(4):1923. doi: 10.3390/ijms27041923 (PMC12940247; doi:10.3390/ijms27041923)
Supplement: Supplementary file 1 [file ijms-27-01923-s001.zip › ijms-4112138-supplementary.pdf]

International Journal of Molecular Sciences

MDPI

Supplementary Material

Table S1. IRS marker expression across Ni categories (II–V): Kruskal–Wallis with post-hoc Mann–Whitney (Bonferroni-adjusted). Values are medians (IRS). Post-hoc p-values are shown as raw p → Bonferroni-adjusted p (p_adj) for significant comparisons (p_adj < 0.05).

Marker	Medians (Ni II → Ni III → Ni IV → Ni V)	KW p	Significant post-hoc (raw p → p_adj)
IL-6	6.0 → 4.0 → 6.0 → 4.0	0.4200	NS
LIF	8.0 → 5.0 → 3.5 → 2.0	<0.001	2 vs 4: 0.0027 → 0.0163 ; 2 vs 5: 1.3×10^{-11}→ 7.9×10^{-11}
LIFR	8.0 → 2.5 → 3.5 → 4.0	<0.001	2 vs 3: 1.1e-05 → 6.4e-05 ; 2 vs 4: 0.0007 → 0.0045 ; 2 vs 5: $1.4\text{x}10^{-8}$ → $8.4\text{x}10^{-8}$
CXCL9	3.0 → 4.0 → 6.0 → 4.0	0.0093	2 vs 4: 0.0016 → 0.0097

Abbreviations: IRS, immunoreactive score; KW, Kruskal–Wallis; Ni, Ni et al. NBI classification.
